# Fasting plasma glucose and HbA1c levels predict the risk of type 2 diabetes and diabetic retinopathy in a Thai high-risk population with prediabetes

**DOI:** 10.3389/fphar.2022.950225

**Published:** 2022-10-04

**Authors:** Chaiwat Washirasaksiri, Weerachai Srivanichakorn, Nutsakol Borrisut, Tullaya Sitasuwan, Rungsima Tinmanee, Chayanis Kositamongkol, Pinyapat Ariyakunaphan, Chonticha Auesomwang, Naruemit Sayabovorn, Thanet Chaisathaphol, Pochamana Phisalprapa

**Affiliations:** ^1^ Department of Medicine, Faculty of Medicine Siriraj Hospital, Mahidol University, Bangkok Noi, Bangkok, Thailand; ^2^ Department of Immunology, Faculty of Medicine Siriraj Hospital, Mahidol University, Bangkok Noi, Bangkok, Thailand

**Keywords:** diabetes incidence, diabetic retinopathy incidence, fasting plasma glucose, high-risk people, HbA1c, prediabetes

## Abstract

**Introduction:** The incidences of diabetes and diabetic retinopathy (DR) in Thai high-risk individuals with prediabetes have not been identified. This study compared diabetes and DR incidences among people at risk with different glycemic levels, using fasting plasma glucose (FPG) and hemoglobin A1C (HbA1c).

**Materials and methods:** A historical cohort study estimating risk of type 2 diabetes and DR was conducted among outpatients, using FPG and HbA1c measurements at recruitment and monitored for ≥5 years. High-risk participants (defined as having metabolic syndrome or atherosclerotic cardiovascular disease) were categorized by glycemic level into 4 groups: 1) impaired fasting glucose (IFG)-/HbA1c- (FPG <110 mg/dl; HbA1c < 6.0%); 2) IFG+/HbA1c- (FPG 110–125 mg/dl; HbA1c < 6.0%); 3) IFG-/HbA1c+ (FPG <110 mg/dl; HbA1c 6.0%–6.4%); and 4) IFG+/HbA1c+ (FPG 110–125 mg/dl; HbA1c 6.0%–6.4%). The incidences of type 2 diabetes mellitus (T2DM) and DR were obtained and estimated using Kaplan-Meier analysis. Cox regression models explored hazard ratios (HRs).

**Results:** We recruited 8,977 people at risk (metabolic syndrome, 89.9%; atherosclerotic cardiovascular disease, 16.9%). The baseline cohort consisted of 1) IFG-/HbA1c- (*n* = 4,221; 47.0%); 2) IFG+/HbA1c- (*n* = 1,274; 14.2%); 3) IFG-/HbA1c+ (*n* = 2,151; 24.0%); and 4) IFG+/HbA1c+ (*n* = 1,331; 14.8%). Their 5-year T2DM incidences were 16.0%, 26.4%, 30.8%, and 48.5% (*p* < 0.001). The median DR follow-up was 7.8 years (interquartile range, 7.0–8.4 years). The DR incidences were 0.50, 0.63, 1.44, and 2.68/1,000 person-years (*p* < 0.001) for IFG-/HbA1c-, IFG+/HbA1c-, IFG-/HbA1c+, and IFG+/HbA1c+, respectively. Compared with IFG-/HbA1c-, the multivariable-adjusted HRs (95% CI) for incident diabetes were 1.94 (1.34–2.80), 2.45 (1.83–3.29), and 4.56 (3.39–6.15) for IFG+/HbA1c-, IFG-/HbA1c+, and IFG+/HbA1c+, respectively. As for incident DR, the corresponding HRs were 0.67 (0.08–5.76), 4.74 (1.69–13.31), and 5.46 (1.82–16.39), respectively.

**Conclusion:** The 5-year incidence of T2DM in Thai high-risk participants with prediabetes was very high. The incidences of diabetes and DR significantly increased with higher degrees of dysglycemia. High-risk people with FPG 110–125 mg/dl and HbA1c 6.0%–6.4% were more likely to develop T2DM and DR. Such individuals should receive priority lifestyle and pharmacological management.

## Introduction

Prediabetes is a high-risk state for diabetes and the associated macrovascular and microvascular complications. According to meta-analyses, approximately 5%–10% of individuals with prediabetes develop diabetes annually. (Gerstein et al., 2007; Tabák et al., 2012) However, the cumulative diabetes incidence varies widely, depending on the population characteristics, definition of prediabetes, duration of follow-up, and study design, ranging from 2% to 50% within a 5-year follow-up. (Zhang et al., 2010; Richter et al., 2018) Moreover, prediabetes is associated with cardiovascular disease, periodontal disease, cognitive dysfunction, fatty liver disease, obstructive sleep apnea, and cancers. (Buysschaert et al., 2015) Prediabetes is generally defined by hyperglycemia above the normal level but below the diabetes threshold. According to the criteria of the World Health Organization, prediabetes can be diagnosed in 2 states: 1) impaired fasting glucose (IFG; fasting plasma glucose [FPG], 110–125 mg/dl) and 2) impaired glucose tolerance (IGT; 2-h postload plasma glucose, 140–199 mg/dl). (World Health Organization, 2006) The International Expert Committee defines “prediabetes” as an HbA1c level of 6.0%–6.4%. (International Expert Committee, 2009)

Prediabetes is a serious healthcare issue affecting one-tenth of the world’s adult population. In 2021, it was estimated that 319 million adults (6.2% of the global population) had IFG, while 541 million adults (10.6% of the global adult population) had IGT. (Sun et al., 2022) The prevalence of prediabetes varies markedly across regions due to underlying factors such as genetic predisposition, obesity, lifestyle, and age. (Ji et al., 2021) Based on IFG and HbA1c analyses, the prediabetes prevalence in the United States is approximately 40% (Centers for Disease Control and Prevention, 2022) and 5%–40% in Asia. (Ji et al., 2021) A high prevalence of prediabetes results in high healthcare expenditure. The economic burden of prediabetes in the United States was determined to exceed 43 billion dollars in 2017. (Dall et al., 2019)

Not all forms of prediabetes are the same. Differences in glycemic parameters and their thresholds result in different diabetes and diabetic retinopathy (DR) rates in people with prediabetes. A meta-analysis of prospective studies between 1979 and 2004 showed that 6%–9% of people with IFG progressed to diabetes annually. (Gerstein et al., 2007) Similarly, a longitudinal study in Japan reported that 7% of individuals with elevated HbA1c progressed to diabetes. (Heianza et al., 2011) A meta-analysis of a multiethnic population demonstrated that prediabetes (defined by an elevated HbA1c between 5.7% and 6.4%) had a higher risk of developing into diabetes than prediabetes defined by IFG. (Lee et al., 2019)

Prediabetes also impacts the progression of DR. Several studies have elucidated the relationship between the chronic hyperglycemia of prediabetes and diabetes and vascular complications (macrovascular and microvascular damage). (Diabetes Prevention Program Research, 2007; Tabak et al., 2012; Ali et al., 2018; Fagg and Valabhji, 2019) Some studies demonstrated that microvascular complications could develop before the standard diabetes diagnosis criteria were met. (Milman and Crandall, 2011) Using the lower FPG and HbA1c cutoffs will increase the apparent prevalence of prediabetes. This move might raise issues for Thailand, given its limited healthcare resources. Moreover, a clear understanding of how different glycemic subgroups affect the incidence of diabetes and its complications is still lacking, especially in Thai high-risk individuals. The present historical cohort study compared the incidences of diabetes and DR and investigated the risks of developing these diseases by assessing FPG and HbA1c in at-risk individuals.

## Materials and methods

Data for this analysis were derived from the medical records of patients who visited the outpatient clinic of Siriraj Hospital, Bangkok, Thailand. The clinic receives self-referrals and referrals from primary and secondary care services. Generally, clinic attendees have a high risk of diabetes or cardiovascular diseases or already have diabetes, hypertension, obesity, or multiple metabolic risk factors. Attendance at the clinic is usually set for regular intervals. Outpatients receive education, appropriate treatments, prevention strategies, and appropriate medications (oral hypoglycemic agents, blood pressure-lowering agents, or cholesterol-lowering agents).

**FIGURE 1:**
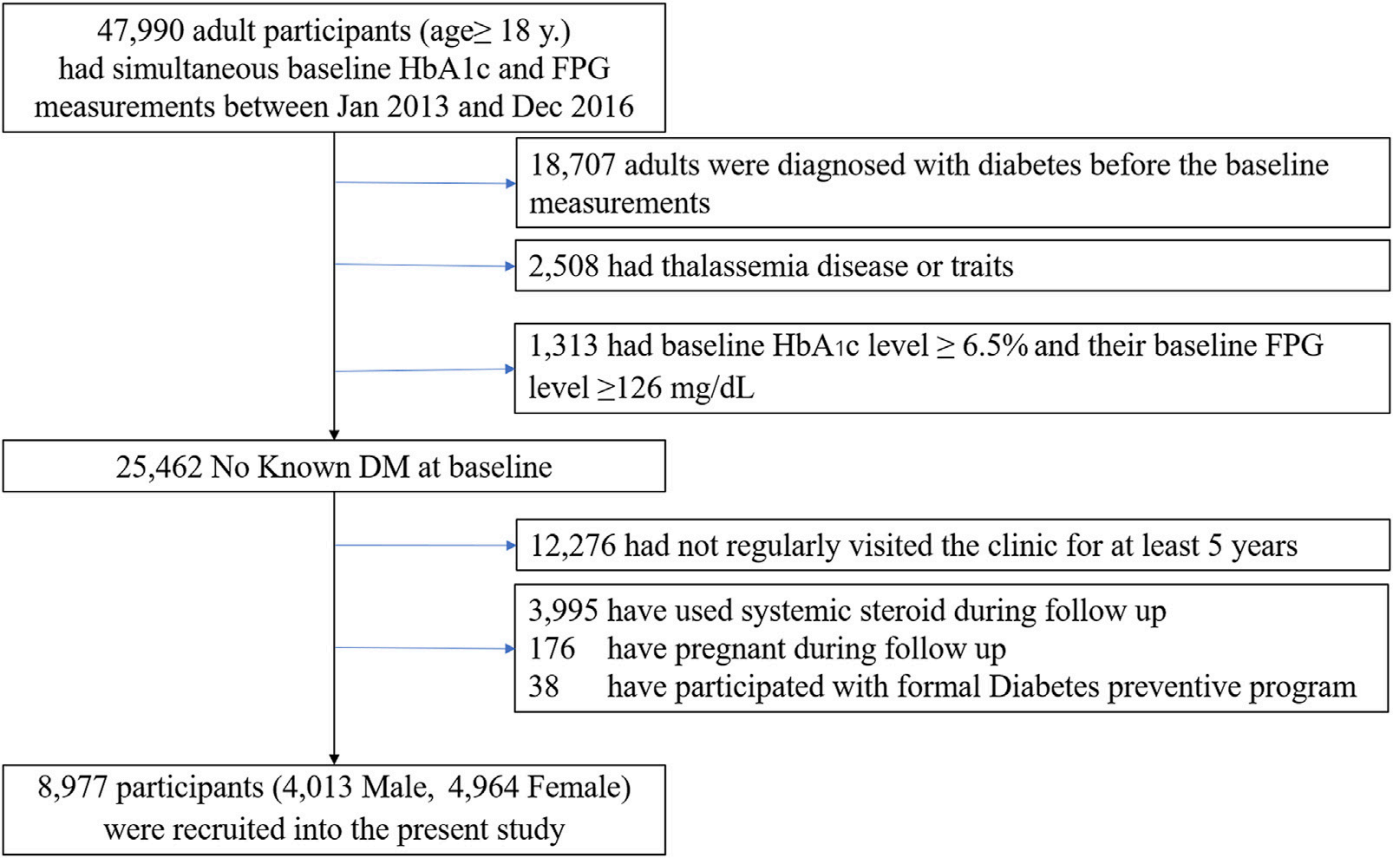
Enrollment of the study subjects.

The dataset for our analysis included people aged at least 18 years at risk of developing diabetes and cardiovascular diseases based on preliminary investigations. The individuals were required to have had simultaneous baseline HbA1c and FPG measurements between January 2013 and December 2016 (*n* = 47,990). Participants were included if 1) their baseline HbA1c level was less than 6.5% (<48 mmol/mol) and 2) their baseline FPG level was under 7.0 mmol/L. Patients were excluded if they met the following criteria (Figure 1):

• were diagnosed with diabetes before the baseline measurements
• had thalassemia disease or traits
• had chronic anemia
• were using any hypoglycemic agent or steroid
• were pregnant

Only subjects with complete data records (age, sex, FPG, HbA1c, and medication use at baseline) who regularly visited the clinic for at least 5 years were included. After exclusions, 8,977 participants without diabetes were available for analysis. The study protocol complied with the Declaration of Helsinki and was reviewed by the Siriraj Institutional Review Board (SI956/2021).

### Procedures and measurements

Clinical characteristics, blood pressure (using an automated measurement system), and anthropometric measurements (height and weight) were collected from electronic health records. “High-risk individuals” were defined as having metabolic syndrome or atherosclerotic cardiovascular disease. “Metabolic syndrome” was defined as participants having at least 3 of the following:

 • body mass index (BMI) ≥ 23 kg/m^2^, per the Asian-specific BMI cutoff (Snehalatha et al., 2003; Consultation', 2004)
 • documented hypertension
 • FPG ≥100 mg/dl or HbA1c ≥ 5.7%
 • a sex-specific low high-density lipoprotein level (males, < 40 mg/dl; females, < 50 mg/dl) or statin use
 •hypertriglyceridemia (fasting triglycerides [TG] ≥ 150 mg/dl) or statin use (Alberti et al., 2005; Alberti et al., 2009)

The blood samples for HbA1c, plasma glucose, and serum lipid measurements were taken in the fasted state. A high-performance liquid chromatography system measured HbA1c with a Tosoh G8 analyzer (Tosoh Bioscience Inc., San Francisco, CA, United States), using the International Federation of Clinical Chemistry-approved methodology and standardization. FPG was measured by an enzymatic hexokinase method. Standard laboratory procedures measured serum total cholesterol, high-density lipoprotein cholesterol (HDL-c), and TG concentrations. Low-density lipoprotein cholesterol (LDL-c) was calculated using the Friedewald formula.

### Outcomes

The primary outcomes were the diabetes incidence and the time to the presence of diabetes within 5 years after the baseline. The secondary outcomes were the DR incidence and the time to DR during a follow-up period of up to 9 years from baseline.

“Diabetes mellitus” (DM) was defined as when patient history met the criteria for DM. These were a diagnosis of DM documented by a doctor, an FPG of 126 mg/dl or higher, an HbA1c of 6.5% or higher, or oral hypoglycemic-agent use. DR was determined by documentation of the condition by a doctor and International Classification of Diseases (10th Revision) coding. The DR documentation was derived from various sources and reports (e.g, ophthalmologist reports of indirect ophthalmoscopy and digital fundus camera findings, documents from other hospitals, and primary doctor’s documentation).

### Statistical analysis

The baseline characteristics of the participants with FPG and HbA1c were compared in these 4 subgroups:

• IFG-/HbA1c-: FPG <110.0 mg/dl (6.1 mmol/L), and HbA1c < 6.0% (<42 mol/mol)
• IFG+/HbA1c-: FPG 110–125 mg/dl (6.1–6.9 mmol/L), and HbA1c < 6.0%
• IFG-/HbA1c+: (HbA1c prediabetes) FPG <110 mg/dl, and HbA1c 6.0%–6.4%
• IFG+/HbA1c+: (combined IFG and HbA1c prediabetes) FPG 110–125 mg/dl, and HbA1c 6.0%–6.4%

Continuous, normally distributed variables are summarized as the mean ± standard deviation, while continuous skewed distribution variables are presented as the median with interquartile ranges. Categorical variables are reported as numbers and percentages. Significant variations across the 4 subgroups were identified by ANOVA (normally distributed continuous variables), the Kruskal–Wallis test (nonnormally distributed variables), and the Chi^2^ test (categorical variables). The incidence rates were estimated using Kaplan-Meier analysis. Cox regression models explored the hazard ratios (HRs) of the DM incidence of each group. Adjustments were made for age, alanine aminotransferase, BMI, hypertensive status, mean corpuscular volume (MCV), sex, statin use, TG level, and uric acid. The HR of DR was explored with adjustments for age, BMI, hypertensive status, MCV, sex, and statin use.

## Results

In all, 8,977 participants were enrolled. There were 4221 IFG-/HbA1c-participants (47.0%), 1274 IFG+/HbA1c-participants (14.2%), 2151 IFG-/HbA1c + participants (24.0%), and 1331 IFG+/HbA1c + participants (14.8%). All subjects had comorbidities, metabolic syndrome, or both. These conditions increase the risk of developing diabetes and atherosclerotic cardiovascular diseases. The participants’ diseases were metabolic syndrome (89.9%), dyslipidemia (70.0%), hypertension (69.8%), and atherosclerotic cardiovascular disease (16.9%). The baseline characteristics of the participants are summarized in Table 1.

**TABLE 1:** Comparison of clinical characteristics of high-risk people in the FPG and HbA1c ranges.

Characteristics	HbA1C <6.0%		HbA1C 6.0%–6.5%		*p* value*
	FPG <110 mg/dl	FPG 110–125 mg/dl	FPG <110 mg/dl	FPG 110–125 mg/dl	
n (8,977)	4,221 (47.0%)	1,274 (14.2%)	2,151 (24.0%)	1,331 (14.8%)	
Female (%)	2,243 (53.1%)	591 (46.5%) ^$^	1,380 (64.2%)	750 (56.3%)	<0.001
Age (years)	59.6 ± 10.6	61.1 ± 10.8^#^	61.4 ± 9.8^#^	61.7 ± 9.7^#^	<0.001
BMI (kg/m2)	25.3 ± 4.0	25.3 ± 4.3	26.2 ± 4.0^#^	26.3 ± 4.0^#^	<0.001
Hypertension (%)	3,339 (79.1%)	1,085 (85.2%)	1813 (84.3%) ^$^	1,161 (87.2%) ^$^	<0.001
Statin use (%)	3,152 (74.7%)	936 (73.5%)	1833 (85.2%) ^$^	1,094 (82.2%) ^$^	<0.001
Metabolic syndrome^#^ (%)	3,632 (86.0%)	1,138 (89.3%) ^$^	2037 (94.7%) ^$^	1,252 (94.1%) ^$^	<0.001
ASCVD (%)	679 (16.1%)	203 (15.9%)	412 (19.2%)	227 (17.1%)	0.01
FPG (mg/dl)	100.7 ± 5.6	114.8 ± 4.0^#^	102.2 ± 4.2^#^	115.4 ± 4.2^#^	<0.001
HbA1C (%)	5.7 ± 0.2	5.7 ± 0.3^&^	6.1 ± 0.1^#^	6.2 ± 0.2^#^	<0.001
Creatinine (mg/dl)	0.91 (0.75–1.08)	0.92 (0.77–1.02) ^#^	0.87 (0.73–1.06) ^$^	0.90 (0.75–1.09) ^#^	<0.001
eGFR (ml/min per 1.73 sq m)	79.5 ± 17.6	79.1 ± 17.9	78.9 ± 17.6	82.3 ± 18.1	0.2
Uric acid (mg/dl)	5.9 ± 1.5	6.2 ± 1.6^&^	6.0 ± 1.4	6.2 ± 1.4^&^	<0.001
Triglycerides (mg/dl)	108 (81–149)	115 (86–161) ^#^	118 (88–159) ^#^	122 (91–168) ^#^	<0.001
Cholesterol (mg/dl)	192.9 ± 40.2	193.9 ± 38.9	191.4 ± 39.5	194.6 ± 40.9	0.1
HDL cholesterol (mg/dl)	57.4 ± 15.9	55.6 ± 15.8^&^	55.8 ± 14.7^&^	53.8 ± 13.5^#^	<0.001
Calculated LDL cholesterol (mg/dl)	111.7 ± 36.1	112.1 ± 35.6	110.8 ± 35.9	115.0 ± 37.1$	0.01
Aspartate transaminase (units/l)	22 (18–27)	22 (19–28)	22 (18–27)	22 (18–28) ^&^	0.003
Alanine aminotransferase (units/l)	21 (15–31)	22 (16–34) ^&^	21 (15–31)	23 (17–33) ^#^	<0.001
Alkaline phosphatase (units/l)	70.7 ± 22.1	76.3 ± 26.0^#^	72.0 ± 21.0	76.0 ± 25.5^#^	<0.001
Gamma-glutamyl transferase (units/l)	30 (20–51)	33 (21–51)	29 (21–50)	32 (22–57)	0.5
MCV	88.8 ± 7.0	89.0 ± 7.4	87.5 ± 7.3^#^	87.9 ± 7.3^$^	<0.001

Data are presented as mean ± standard deviation, n (%), or median (25th–75th percentile). ^#^ Metabolic syndrome was defined as having at least 3 out of 5 of these criteria: 1) body mass index >23 kg/m^2^, according to Asian-specific BMI, cutoffs; 2) documented hypertension; 3) FPG ≥100 mg/dl or HbA1c ≥ 5.7%; 4) sex-specific, low-HDL, criteria (male <40 mg/dl or female <50 mg/dl) or statin use; 5) hypertriglyceridemia (fasting TG ≥ 150 mg/dl) or statin use.

Categorical variable, percentage (number); continuous normally distributed variable, mean ± standard deviation or continuous skewed distribution variable; median and interquartile range are shown.

ASCVD, atherosclerotic cardiovascular disease; BMI, body mass index; eGRF, estimated glomerular filtration rate; FPG, fasting plasma glucose; HbA1c, hemoglobin A1c; HDL, high-density lipoprotein; kg/m2, kilogram per square meter; LDL, low-density lipoprotein; MCV, mean corpuscular volume; mg/dl, milligram per deciliter; mmHg, millimeters mercury; mmol/L, millimole per liter.

Blood samples for HbA1c, plasma glucose, serum uric acid, and serum lipid measurements were taken in the fasted state. Serum aspartate transaminase, alanine aminotransferase, alkaline phosphatase, gamma-glutamyl transferase, serum creatinine, and blood for MCV were taken in either the fasted or nonfasted state.

* Significant variations across glycemic categories were identified for normally distributed continuous variables by ANOVA, for non-normally distributed variables by Kruskal–Wallis non-parametric ANOVA, and for categorical variables by Chi^2^ test. Bonferroni adjustment for multiple testing:^#^ *p* < 0.001, ^&^ *p* < 0.01, ^$^ *p* < 0.05 compared with HbA1c < 6.0% and FPG <110 mg/dl.

With increasing degrees of glucose homeostasis in the participants with prediabetes, significant increases were found in age, alkaline phosphatase, BMI, the proportion of people with hypertension, statin use, and TG. However, the participants’ HDL-c levels fell significantly. All changes were especially noticeable among subjects with HbA1c levels between 6.0% and 6.4%.

### Progression to diabetes

By the 5-year follow-up, 2,319 subjects (25.8%) had developed diabetes. The incidence of diabetes varied significantly among the prediabetes subgroups (*p* < 0.001). The IFG+/HbA1c + subgroup had the highest incidence rate (48.5%), followed by the IFG-/HbA1c + subgroup (30.8%), the IFG+/HbA1c-subgroup (26.4%), and the IFG-/HbA1c-subgroup (16.0%). The adjusted multivariable Cox regression model found that subjects in the IFG+/HbA1c+, IFG-/HbA1c+, and IFG+/HbA1c-subgroups had significantly higher risks of 5-year diabetes incidence than subjects in the IFG-/HbA1c-subgroup. The HRs of the first 3 subgroups were 4.56 (95% CI, 3.39–6.15), 2.45 (95% CI, 1.83–3.29), and 1.94 (95% CI, 1.34–2.80), respectively (Table 2). Moreover, patients with a BMI above 30.0 kg/m^2^, hypertension, and statin use had a significantly increased risk of diabetes conversion than 1) those with a BMI <23 kg/m^2^ (HR, 1.87; 95% CI, 1.29–2.71), 2) those without hypertension (HR, 1.90; 95% CI, 1.36–2.66), and 3) those who did not use statins (HR, 2.02; 95% CI, 1.46–2.89; Supplementary Table S1).

**TABLE 2:** Five-year incidence and hazard ratios for the development of diabetes by baseline prediabetes subgroup.

Prediabetes subgroups	5-year diabetes outcomes		HR (95% CI)*
	DM incidence n (%)	Non-progression n (%)	
HbA1C < 6.0%			
FPG <110 mg/dl	675 (16.0%)	3,546 (84.0%)	1
FPG 110–125 mg/dl	336 (26.4%)	938 (73.6%)	1.94 (1.34–2.80)
HbA1C 6.0%–6.5%			
FPG <110 mg/dl	663 (30.8%)	1,488 (69.2%)	2.45 (1.83–3.29)
FPG 110–125 mg/dl	645 (48.5%)	686 (51.5%)	4.56 (3.39–6.15)
2,319 (25.8%)	2,319 (25.8%)	6,658 (74.2%)	

CI, confidence interval; DM, diabetes mellitus; FPG, fasting plasma glucose; HbA1C, glycated hemoglobin A1c; HR, hazard ratio.

* Hazard ratios of type 2 diabetes mellitus incidence in each glycemic range were explored by Cox regression models with adjustments for age, alanine aminotransferase, body mass index, hypertensive status, mean corpuscular volume, sex, statin use, triglyceride level, and uric acid.

### Progression to diabetic retinopathy

The 8,977 patients contributed to 68,643 person-years with a median follow-up duration of 7.8 years (IQR, 7.0–8.4 years). The participants with IFG+/HbA1c + had the highest incidence rate of DR (2.68 per 1,000 person-years). The other rates were IFG-/HbA1c+, 1.44 per 1,000 person-years; IFG+/HbA1c-, 0.6 per 1,000 person-years; and IFG-/HbA1c-, 0.5 per 1,000 person-years (*p* = 0.001). The adjusted multivariable Cox regression model determined that compared with the IFG-/HbA1c-subgroup, the adjusted HRs were 5.46 (95% CI, 1.82–16.39) for the IFG+/HbA1c + subgroup, 4.73 (95% CI, 1.69–13.31) for IFG-/HbA1c+, and 0.67 (95% CI, 0.08–5.76) for IFG+/HbA1c- (Table 3). In this adjusted regression model, age, BMI category, hypertension, MCV, sex, and statin status were not associated with DR risk (Supplementary Table S2).

**TABLE 3:** Incidence and hazard ratios for the development of diabetic retinopathy by baseline prediabetes subgroup.

Prediabetes subgroups	Total	DR outcomes		HR* (95% CI)
		Incident cases/person-year	Incident rate/1,000 person-years	
HbA1C < 6.0%				
FPG <110 mg/dl	4,221	16/32,352	0.50	1
FPG 110–125 mg/dl	1,274	6/9,570	0.63	0.67 (0.08–5.76)
HbA1C 6.0%–6.5%				
FPG <110 mg/dl	2,151	24 (16,648)	1.44	4.73 (1.69–13.31)
FPG 110–125 mg/dl	1,331	27 (10,073)	2.68	5.45 (1.82–16.39)
Total	8,977	73 (68,643)	1.1	

CI, confidence interval; DR, diabetic retinopathy; FPG, fasting plasma glucose; HbA1C, glycated hemoglobin A1c; HR, hazard ratio.

* Hazard ratios of diabetic retinopathy were explored with adjustments for age, body mass index, hypertensive status, mean corpuscular volume, sex, and statin use.

## Discussion

Several studies have reported adverse associations between nondiabetic hyperglycemia and the incidence of diabetes in general populations. However, the present study is among the few to demonstrate that these associations in Thai individuals with prediabetes signal high risk for diabetes and cardiovascular disease. We found that rising glycemia resulting from deteriorating glucose homeostasis was associated with worsening cardiometabolic risks, particularly in individuals with more elevated HbA1c levels. Additionally, the incidences of diabetes and DR increased substantially with the severity of FPG and HbA1c abnormalities. The combination of IFG and HbA1c prediabetes carried the highest risk of developing diabetes and DR (4.56 and 5.46 times, compared with IFG-/HbA1c-, for developing diabetes and DR, respectively).

Previous international studies examining the progression from prediabetes to diabetes found that people with prediabetes had different risks of developing diabetes, depending on their glucose level in the prediabetes range. (Gerstein et al., 2007; Zhang et al., 2010; Echouffo-Tcheugui and Selvin, 2021) This finding is consistent with a meta-analysis of prospective cohort studies that showed that individuals with the combination of more than one indicator of prediabetes (IFG+/IGT+) had the highest risk of developing diabetes. (Richter et al., 2018) The current investigation’s results correlate with those of a previous study in Thailand. That research demonstrated that the highest accuracy for progression to diabetes in participants with prediabetes (defined by IGT or IFG at baseline) was a combination of FPG levels above 110 mg/dl and HbA1c levels above 6.0%. (Wutthisathapornchai A 2021)

Most research into the progression from prediabetes to diabetes has examined the relationship using 2-h postload plasma glucose or FPG as prediabetes criteria in general individuals. However, the current work focused on high-risk prediabetes categorized by HbA1c and FPG. HbA1c represents the average blood glucose level during approximately the preceding 3 months (Nathan et al., 2007b; Syed, 2011) and signifies the risk for diabetes. HbA1c is used worldwide to estimate glycemic control, whereas the glucose tolerance test is limited in use because it is time-consuming. The present investigation provides information about a practical glycemic evaluation method that draws upon HbA1c and FPG.

On the other hand, an impairment in FPG indicates a decrease in hepatic insulin sensitivity and a defect in early-phase insulin secretion. (Incani et al., 2015; Aoyama-Sasabe et al., 2016) A finding of IGT denotes a decline in insulin sensitivity in muscles and a defect in late-phase insulin secretion. (Nathan et al., 2007a) Moreover, HbA1c prediabetes results from a combination of IFG and IGT. (Faerch et al., 2015; Incani et al., 2015) Although a meta-analysis demonstrated that people with isolated IGT and individuals with the combination of IGT and IFG had a high relative risk for future diabetes (Gerstein et al., 2007), measuring IGT can be challenging in clinical practice and epidemiological studies. Given these considerations, disturbances in glucose homeostasis in people with both IFG and HbA1c also likely signify a high risk of progressing to diabetes, with several advantages in clinical practice.

Our model established that hypertension status and statin use significantly increased the risk of diabetes conversion. The expected relationships between either hypertension status or statin use and diabetes incidence were confirmed. In high-risk people with prediabetes, the antihypertensive drug class, blood-pressure-lowering potency of the drug used, and the type of statin may affect these relationships, as revealed by previous meta-analyses. (Sattar et al., 2010; Nazarzadeh et al., 2021) However, as our dataset lacked information on the medication type, the group of antihypertensive agents, and statin use, we could not fully explore the relationships.

The early stages of retinal complications, renal involvement, and neurological damage were reported in people with prediabetes. (Tabak et al., 2012; Stino and Smith, 2017; Ali et al., 2018) A critical and clinically relevant finding is that, during a median follow-up of 7.8 years, the DR incidence rose from 0.50/1,000 person-years to a peak of 2.68/1,000 person-years in people with both IFG and HbA1c prediabetes. The research confirms the association between prediabetes and microvascular complications observed in earlier data extracted from the Diabetes Prevention Program in the United States. Data analysis revealed that approximately 8% of participants with IGT had DR. (Diabetes Prevention Program Research, 2007) The association between IGT and DR also correlated with other studies demonstrating that DR incidence rose linearly with an increase in blood glucose (especially HbA1c levels). (Cheng et al., 2009; Lee et al., 2019) Overall, the various findings support the proposition that microvascular complications, particularly DR, involve gradual and continuous processes triggered by chronic exposure to glucose. Moreover, microvascular complications may occur before diabetes is clinically diagnosable.

Our investigation demonstrated that an IFG between 110 and 126 mg/dl was not an independent risk factor for progression to DR. A further parallel with this observation was reported by a study assessing the connection between DR prevalence and glycemia in an adult population aged ≥40 years. That work detected a rapid increase in DR prevalence with rising glycemia and found that HbA1c was a better predictive factor than FPG. (Cheng et al., 2009) Furthermore, a similar relationship between IFG and cardiovascular disease was found as impaired fasting glucose carried a lower risk of cardiovascular disease than impaired glucose tolerance. (Cai et al., 2020) Other studies have reported that IFG was not an independent risk factor for cardiovascular disease. (DECODE Study Group, 1999)

As for diabetes prevention, dietary control and regular exercise should generally be recommended to individuals with prediabetes. Supplementary intensive lifestyle or pharmacological interventions can increase the effectiveness of basic lifestyle changes and are cost-effective, especially in individuals with IGT. (Pan et al., 1997; Knowler et al., 2002; Lindstrom et al., 2003; Wylie-Rosett et al., 2006) Research has also shown that intensive lifestyle intervention in people with IGT can decrease the incidence of DR compared with routine care. (Aro et al., 2019; Gong et al., 2019) A meta-analysis demonstrated that high-risk individuals with prediabetes—particularly those with atherosclerotic cardiovascular diseases—have significantly higher long-term risks of composite cardiovascular disease and total death than individuals with normal glucose regulation. (Hostalek and Campbell, 2021)

On the other hand, additional lifestyle and pharmacological measures are costly, time-consuming, and require much effort by individuals and healthcare professionals conducting group and monitoring interventions. (Knowler et al., 2002) Moreover, previous meta-analyses have revealed an increase in the risk of type 2 diabetes with lipid-lowering treatments and some antihypertensive drugs through a blood pressure lowering effect as well as off-target mechanisms, which are the primary treatments in atherosclerotic cardiovascular disease. The current investigation helps by profiling risk levels, enabling clinicians to identify high-risk individuals with prediabetes to be persuaded to engage in intensive lifestyle interventions or to use medications, including appropriate antihypertensive class and statin type. (Sattar et al., 2010; Nazarzadeh et al., 2021) Such individuals are those with both IFG and HbA1c prediabetes (per the World Health Organization criteria and International Expert Committee, respectively), given that the present study found they have the highest risk of developing diabetes and DR.

Our analysis has its strengths. This research is the first to examine the relationships between glycemic range and cardiometabolic risk factors, diabetes, and DR in Thai high-risk individuals with prediabetes. In contrast, the previously mentioned Thai studies only provided information on DM incidence in the low-to moderate-risk population with prediabetes. (Thamakaison et al., 2021; Wutthisathapornchai A 2021) In addition, the analyses drew on an extensive study sample.

Our study also has limitations. Perhaps the most fundamental limitation is that our data were derived from a retrospective cohort constructed from databases of healthcare records. The cohort may not represent all possible people with prediabetes in the general population, health records may not have all pertinent risk factors, and diagnoses are likely to have been identified but not subsequently recorded wholly and correctly. Additionally, the DR diagnoses were obtained from various sources and reports. These DR documentation findings depend on the skill and experience of the attending ophthalmologist, and the ophthalmological devices and procedures used. These limitations may have biased the observed associations.

However, in our analysis, the exposure to all relevant risk factors was recorded before the occurrence of diabetes and DR. Consequently, the temporal sequence of risk factors and outcomes could be assessed. The diagnoses and definitions of all related risk factors were defined carefully using standard references and all relevant record parameters (medication records, International Classification of Diseases (10th Revision) coding, and laboratory findings). The diagnoses and risk factors were further reviewed by trained research staff. Last, our model analysis did not include the IGT measurement because, in Thailand, we do not routinely measure IGT in clinical practice. Consequently, there were few data on IGT in our dataset.

In conclusion, people with prediabetes are at high risk of developing diabetes in the short term, and the risk of progression depends on the criteria of prediabetes and the number of pathogeneses. Moreover, diabetic retinal complications can be found in people with prediabetes. Using the World Health Organization criteria, this study revealed that people with prediabetes who had an FPG of 110–125 mg/dl or an HbA1c of 6.0%–6.4% were likely to develop type 2 DM and DR. Early intensive intervention and pharmacological treatment should be considered for these high-risk subgroups. Further randomized control trials are needed to confirm the benefits of these interventions in high-risk people with prediabetes, especially those with the combination of FPG and HbA1c prediabetes.

## Data Availability

The original contributions presented in the study are included in the article/Supplementary Material, further inquiries can be directed to the corresponding author.

## References

[B1] Alberti K. G. Eckel R. H. Grundy S. M. Zimmet P. Z. Cleeman J. I. Donato K. A. (2009). Harmonizing the metabolic syndrome: A joint interim statement of the international diabetes federation task force on epidemiology and prevention; national heart, lung, and blood Institute; American heart association; world heart federation; international atherosclerosis society; and international association for the study of obesity. Circulation 120 (16), 1640–1645. 10.1161/CIRCULATIONAHA.109.192644 19805654

[B2] Alberti K. G. Zimmet P. Shaw J. Group I. D. F. E. T. F. C. (2005). The metabolic syndrome-a new worldwide definition. Lancet 366 (9491), 1059–1062. 10.1016/S0140-6736(05)67402-8 16182882

[B3] Ali M. K. Bullard K. M. Saydah S. Imperatore G. Gregg E. W. (2018). Cardiovascular and renal burdens of prediabetes in the USA: Analysis of data from serial cross-sectional surveys, 1988-2014. Lancet. Diabetes Endocrinol. 6 (5), 392–403. 10.1016/S2213-8587(18)30027-5 29500121 PMC6615033

[B4] Aoyama-Sasabe S. Fukushima M. Xin X. Taniguchi A. Nakai Y. Mitsui R. (2016). Insulin secretory defect and insulin resistance in isolated impaired fasting glucose and isolated impaired glucose tolerance. J. Diabetes Res. 2016, 1298601. 10.1155/2016/1298601 26788515 PMC4693016

[B5] Aro A. Kauppinen A. Kivinen N. Selander T. Kinnunen K. Tuomilehto J. (2019). Life style intervention improves retinopathy status—the. Finn. Diabetes Prev. Study 11 (7), 1691. 10.3390/nu11071691 PMC6683279 31340493

[B6] Buysschaert M. Medina J. L. Bergman M. Shah A. Lonier J. (2015). Prediabetes and associated disorders. Endocrine 48 (2), 371–393. 10.1007/s12020-014-0436-2 25294012

[B7] Cai X. Zhang Y. Li M. Wu J. H. Mai L. Li J. (2020). Association between prediabetes and risk of all cause mortality and cardiovascular disease: Updated meta-analysis. Bmj 370, m2297. 10.1136/bmj.m2297 32669282 PMC7362233

[B8] Centers for Disease Control and Prevention (2022). National diabetes statistics report. Available: https://www.cdc.gov/diabetes/data/statistics-report/index.html.

[B9] Cheng Y. J. Gregg E. W. Geiss L. S. Imperatore G. Williams D. E. Zhang X. (2009). Association of A1C and fasting plasma glucose levels with diabetic retinopathy prevalence in the U.S. population: Implications for diabetes diagnostic thresholds. Diabetes Care 32 (11), 2027–2032. 10.2337/dc09-0440 19875604 PMC2768189

[B10] Consultation' W. H. O. E. (2004). Appropriate body-mass index for Asian populations and its implications for policy and intervention strategies. Lancet 363 (9403), 157–163. 10.1016/S0140-6736(03)15268-3 14726171

[B11] Dall T. M. Yang W. Gillespie K. Mocarski M. Byrne E. Cintina I. (2019). The economic burden of elevated blood glucose levels in 2017: Diagnosed and undiagnosed diabetes, gestational diabetes mellitus, and prediabetes. Diabetes care 42 (9), 1661–1668. 10.2337/dc18-1226 30940641 PMC6702607

[B12] DECODE Study Group (1999). New diagnostic criteria for diabetes and mortality in older adults. DECODE Study Group. European Diabetes Epidemiology Group. Lancet 353 (9146), 68–69. 10.1016/s0140-6736(05)74840-6 10023974

[B13] Diabetes Prevention Program Research (2007). The prevalence of retinopathy in impaired glucose tolerance and recent-onset diabetes in the Diabetes Prevention Program. Diabet. Med. 24 (2), 137–144. 10.1111/j.1464-5491.2007.02043.x 17257275 PMC2267935

[B14] Echouffo-Tcheugui J. B. Selvin E. (2021). Prediabetes and what it means: The epidemiological evidence. Annu. Rev. Public Health 42, 59–77. 10.1146/annurev-publhealth-090419-102644 33355476 PMC8026645

[B15] Faerch K. Johansen N. B. Witte D. R. Lauritzen T. Jorgensen M. E. Vistisen D. (2015). Relationship between insulin resistance and beta-cell dysfunction in subphenotypes of prediabetes and type 2 diabetes. J. Clin. Endocrinol. Metab. 100 (2), 707–716. 10.1210/jc.2014-2853 25387263

[B16] Fagg J. Valabhji J. (2019). How do we identify people at high risk of Type 2 diabetes and help prevent the condition from developing? Diabet. Med. 36 (3), 316–325. 10.1111/dme.13867 30466172 PMC6590463

[B17] Gerstein H. C. Santaguida P. Raina P. Morrison K. M. Balion C. Hunt D. (2007). Annual incidence and relative risk of diabetes in people with various categories of dysglycemia: A systematic overview and meta-analysis of prospective studies. Diabetes Res. Clin. Pract. 78 (3), 305–312. 10.1016/j.diabres.2007.05.004 17601626

[B18] Gong Q. Zhang P. Wang J. Ma J. An Y. Chen Y. (2019). Morbidity and mortality after lifestyle intervention for people with impaired glucose tolerance: 30-year results of the da qing diabetes prevention outcome study. Lancet. Diabetes Endocrinol. 7 (6), 452–461. 10.1016/S2213-8587(19)30093-2 31036503 PMC8172050

[B19] Heianza Y. Hara S. Arase Y. Saito K. Fujiwara K. Tsuji H. (2011). HbA1c 5· 7–6· 4% and impaired fasting plasma glucose for diagnosis of prediabetes and risk of progression to diabetes in Japan (TOPICS 3): A longitudinal cohort study. Lancet 378 (9786), 147–155. 10.1016/S0140-6736(11)60472-8 21705064

[B20] Hostalek U. Campbell I. (2021). Metformin for diabetes prevention: Update of the evidence base. Curr. Med. Res. Opin. 37 (10), 1705–1717. 10.1080/03007995.2021.1955667 34281467

[B21] Incani M. Sentinelli F. Perra L. Pani M. G. Porcu M. Lenzi A. (2015). Glycated hemoglobin for the diagnosis of diabetes and prediabetes: Diagnostic impact on obese and lean subjects, and phenotypic characterization. J. Diabetes Investig. 6 (1), 44–50. 10.1111/jdi.12241 PMC4296702 25621132

[B22] International Expert Committee (2009). International Expert Committee report on the role of the A1C assay in the diagnosis of diabetes. Diabetes care 32 (7), 1327–1334. 10.2337/dc09-9033 19502545 PMC2699715

[B23] Ji L. Pranoto A. Andag-Silva A. Deerochanawong C. Van Phuoc D. Tan K. C. B. (2021). Western pacific consensus proposals for management of prediabetes. Int. J. Clin. Pract. 75, e14019. 10.1111/ijcp.14019 33480067

[B24] Knowler W. C. Barrett-Connor E. Fowler S. E. Hamman R. F. Lachin J. M. Walker E. A. (2002). Reduction in the incidence of type 2 diabetes with lifestyle intervention or metformin. N. Engl. J. Med. 346 (6), 393–403. 10.1056/NEJMoa012512 11832527 PMC1370926

[B25] Lee C. M. Y. Colagiuri S. Woodward M. Gregg E. W. Adams R. Azizi F. (2019a). Comparing different definitions of prediabetes with subsequent risk of diabetes: An individual participant data meta-analysis involving 76 513 individuals and 8208 cases of incident diabetes. BMJ Open Diabetes Res. Care 7 (1), e000794. 10.1136/bmjdrc-2019-000794 PMC6936411 31908797

[B26] Lee M. Y. Hsu W. H. Lai C. W. Chen S. C. Liang C. C. (2019b). The association between glycated albumin, glycohemoglobin, and glycated albumin to glycohemoglobin ratio in diabetic retinopathy of prediabetes. Kaohsiung J. Med. Sci. 35 (11), 695–701. 10.1002/kjm2.12125 31483568 PMC11900673

[B27] Lindstrom J. Louheranta A. Mannelin M. Rastas M. Salminen V. Eriksson J. (2003). The Finnish Diabetes Prevention Study (DPS): Lifestyle intervention and 3-year results on diet and physical activity. Diabetes Care 26 (12), 3230–3236. 10.2337/diacare.26.12.3230 14633807

[B28] Milman S. Crandall J. P. (2011). Mechanisms of vascular complications in prediabetes. Med. Clin. North Am. 95 (2), 309–325. vii. 10.1016/j.mcna.2010.11.004 21281835

[B29] Nathan D. M. Davidson M. B. DeFronzo R. A. Heine R. J. Henry R. R. Pratley R. (2007a). Impaired fasting glucose and impaired glucose tolerance: Implications for care. Diabetes Care 30 (3), 753–759. 10.2337/dc07-9920 17327355

[B30] Nathan D. M. Turgeon H. Regan S. (2007b). Relationship between glycated haemoglobin levels and mean glucose levels over time. Diabetologia 50 (11), 2239–2244. 10.1007/s00125-007-0803-0 17851648 PMC8752566

[B31] Nazarzadeh M. Bidel Z. Canoy D. Copland E. Wamil M. Majert J. (2021). Blood pressure lowering and risk of new-onset type 2 diabetes: An individual participant data meta-analysis. Lancet 398 (10313), 1803–1810. 10.1016/S0140-6736(21)01920-6 34774144 PMC8585669

[B32] Pan X.-R. Li G.-W. Hu Y.-H. Wang J.-X. Yang W.-Y. An Z.-X. (1997). Effects of diet and exercise in preventing NIDDM in people with impaired glucose tolerance. The Da Qing IGT and Diabetes Study. Diabetes Care 20 (4), 537–544. 10.2337/diacare.20.4.537 9096977

[B33] Richter B. Hemmingsen B. Metzendorf M. I. Takwoingi Y. (2018). Development of type 2 diabetes mellitus in people with intermediate hyperglycaemia. Cochrane Database Syst. Rev. 10, CD012661. 10.1002/14651858.CD012661.pub2 30371961 PMC6516891

[B34] Sattar N. Preiss D. Murray H. M. Welsh P. Buckley B. M. de Craen A. J. (2010). Statins and risk of incident diabetes: A collaborative meta-analysis of randomised statin trials. Lancet 375 (9716), 735–742. 10.1016/S0140-6736(09)61965-6 20167359

[B35] Snehalatha C. Viswanathan V. Ramachandran A. (2003). Cutoff values for normal anthropometric variables in Asian Indian adults. Diabetes Care 26 (5), 1380–1384. 10.2337/diacare.26.5.1380 12716792

[B36] Stino A. M. Smith A. G. (2017). Peripheral neuropathy in prediabetes and the metabolic syndrome. J. Diabetes Investig. 8 (5), 646–655. 10.1111/jdi.12650 PMC5583955 28267267

[B37] Sun H. Saeedi P. Karuranga S. Pinkepank M. Ogurtsova K. Duncan B. B. (2022). IDF Diabetes Atlas: Global, regional and country-level diabetes prevalence estimates for 2021 and projections for 2045. Diabetes Res. Clin. Pract. 183, 109119. 10.1016/j.diabres.2021.109119 34879977 PMC11057359

[B38] Syed I. A. (2011). Glycated haemoglobin; past, present, and future are we ready for the change. J. Pak. Med. Assoc. 61 (4), 383–388. 21465979

[B39] Tabak A. G. Herder C. Rathmann W. Brunner E. J. Kivimaki M. (2012). Prediabetes: A high-risk state for diabetes development. Lancet 379 (9833), 2279–2290. 10.1016/S0140-6736(12)60283-9 22683128 PMC3891203

[B40] Thamakaison S. Anothaisintawee T. Sukhato K. Unwanatham N. Rattanasiri S. Reutrakul S. (2021). Hemoglobin A1c in combination with fasting plasma glucose trumps fasting plasma glucose alone as predictive indicators for diabetes mellitus: An ambidirectional cohort study of Thai people with impaired fasting glucose. BMJ Open Diabetes Res. Care 9 (2), e002427. 10.1136/bmjdrc-2021-002427 PMC8634022 34845059

[B42] World Health Organization (2006). Definition and diagnosis of diabetes mellitus and intermediate hyperglycaemia: Report of a WHO/IDF consultation. Geneva, Switzerland: WHO.

[B43] Wutthisathapornchai A L. R. (2021). Progression of prediabetes to type 2 diabetes mellitus in Thai population. J. Med. Assoc. Thai 104, 12099. 10.35755/jmedassocthai.2021.05.12099

[B44] Wylie-Rosett J. Herman W. H. Goldberg R. B. (2006). Lifestyle intervention to prevent diabetes: Intensive and cost effective. Curr. Opin. Lipidol. 17 (1), 37–44. 10.1097/01.mol.0000203890.27267.eb 16407714

[B45] Zhang X. Gregg E. W. Williamson D. F. Barker L. E. Thomas W. Bullard K. M. (2010). A1C level and future risk of diabetes: A systematic review. Diabetes Care 33 (7), 1665–1673. 10.2337/dc09-1939 20587727 PMC2890379

