# Supplementary material for: Fasting plasma glucose and HbA1c levels predict the risk of type 2 diabetes and diabetic retinopathy in a Thai high-risk population with prediabetes
Source: Front Pharmacol. 2022 Oct 4;13:950225. doi: 10.3389/fphar.2022.950225 (PMC9576996; doi:10.3389/fphar.2022.950225)
Supplement: Supplementary file 1 [file DataSheet1.docx]

| **Supplementary Table 1.** 5-year incidence and hazard ratios for development of diabetes: independent influences of age, alanine aminotransferase, BMI, hypertensive status, mean corpuscular volume, sex, statin use, triglyceride level, and uric acid | | | | | | | | | |  |
| --- | --- | --- | --- | --- | --- | --- | --- | --- | --- | --- |
| **Covariate** | | | **Coefficient** | | **Standard error** | ***P* value** | **HR** | **95% CI** | |  |
|  |  |  |  |  |  |  |  | **Lower** | **Upper** |  |
| Age (years) | | | -0.010 |  | 0.006 | 0.082 | 0.99 | 0.978 | 1.001 |  |
| Sex | | | 0.085 |  | 0.127 | 0.502 | 1.089 | 0.85 | 1.395 |  |
| BMI (kg/m2)* | | |  |  |  |  |  |  |  |  |
|  | Normal | : BMI less than 23 |  |  |  |  |  |  |  |  |
|  | Overweight | : BMI 23.0–24.9 | -0.161 |  | 0.191 | 0.398 | 0.851 | 0.586 | 1.237 |  |
|  | Obese I | : BMI 25.0–29.9 | 0.145 |  | 0.159 | 0.362 | 1.156 | 0.846 | 1.580 |  |
|  | Obese II | : BMI more than 30.0 | 0.627 |  | 0.189 | 0.001 | 1.873 | 1.294 | 2.711 |  |
| Hypertension status | | | 0.641 |  | 0.171 | < 0.001 | 1.898 | 1.357 | 2.655 |  |
| Statin status | | | 0.702 |  | 0.164 | < 0.001 | 2.017 | 1.462 | 2.783 |  |
| Prediabetes subgroups** | | |  |  |  |  |  |  |  |  |
|  | IFG-/HbA1c- | |  |  |  |  |  |  |  |  |
|  | IFG+/HbA1C- | | 0.662 |  | 0.188 | < 0.001 | 1.939 | 1.341 | 2.803 |  |
|  | IFG-/HbA1C+ | | 0.898 |  | 0.149 | < 0.001 | 2.454 | 1.833 | 3.287 |  |
|  | IFG+/HbA1C+ | | 1.518 |  | 0.152 | < 0.001 | 4.563 | 3.385 | 6.151 |  |
| Triglyceride (mg/dl) | | | 0.001 |  | 0.001 | 0.209 | 1.001 | 0.999 | 1.002 |  |
| Alanine aminotransferase (units/l) | | | 0.003 |  | 0.002 | 0.125 | 1.003 | 0.999 | 1.008 |  |
| Uric acid (mg/dl) | | | 0.075 |  | 0.042 | 0.07 | 1.078 | 0.994 | 1.169 |  |
| MCV | | | -0.004 |  | 0.008 | 0.653 | 0.996 | 0.98 | 1.013 |  |
| BMI, body mass index; CI, confidence interval; HbA1C, glycated hemoglobin A1c; HR, hazard ratio; IFG, impaired fasting glucose; kg/m2, kilogram per square meter; MCV, mean corpuscular volume; mg/dl, milligram per deciliter  * BMI criteria by WHO Asia-Pacific criteria  ** (1) IFG-/HbA1c-: FPG < 110.0 mg/dL (6.1 mmol/L) and HbA1c < 6.0% (< 42 mmol/mol); (2) IFG+/HbA1c-: FPG 110–125 mg/dL (6.1–6.9 mmol/L) and HbA1c < 6.0%; (3) HbA1c prediabetes (IFG-/HbA1c+): FPG < 110 mg/dL and HbA1c 6.0%–6.4%; (4) combined IFG and HbA1c prediabetes (IFG+/HbA1c+): FPG 110–125 mg/dL and HbA1c 6.0%–6.4%  *Hazard ratios of type 2 diabetes mellitus incidence in each glycemic range were explored by Cox regression models with adjustments for age, alanine aminotransferase, body mass index, hypertensive status, mean corpuscular volume, sex, statin use, triglyceride level, and uric acid. | | | | | | | | | | |

| **Supplementary Table 2.** Incidence and hazard ratios for development of diabetic retinopathy: independent influences of age, body mass index, hypertensive status, mean corpuscular volume, sex, and statin use | | | | | | | | | |
| --- | --- | --- | --- | --- | --- | --- | --- | --- | --- |
| **Covariate** | | | **Coefficient** | | **Standard error** | ***P* value** | **HR** | **95% CI** | |
|  |  |  |  |  |  |  |  | **Lower** | **Upper** |
| Age (years) | | | 0.030 |  | 0.020 | 0.122 | 1.031 | 0.992 | 1.071 |
| Gender | | | -0.039 |  | 0.383 | 0.919 | 0.962 | 0.454 | 2.038 |
| BMI (kg/m2) * | | |  |  |  |  |  |  |  |
|  | Normal | : BMI less than 23 |  |  |  |  |  |  |  |
|  | Overweight | : BMI 23.0–24.9 | 0.376 |  | 0.767 | 0.624 | 1.456 | 0.324 | 6.552 |
|  | Obese I | : BMI 25.0–29.9 | 1.061 |  | 0.639 | 0.097 | 2.889 | 0.826 | 10.110 |
|  | Obese II | : BMI more than 30.0 | 1.300 |  | 0.726 | 0.073 | 3.669 | 0.884 | 15.218 |
| Hypertension status | | | 0.625 |  | 0.751 | 0.405 | 1.868 | 0.429 | 8.141 |
| Statin status | | | 0.468 |  | 0.619 | 0.450 | 1.596 | 0.474 | 5.375 |
| Prediabetes subgroups** | | |  |  |  |  |  |  |  |
|  | IFG-/HbA1C- | |  |  |  |  |  |  |  |
|  | IFG+/HbA1C- | | -0.398 |  | 1.096 | 0.716 | 0.671 | 0.078 | 5.759 |
|  | IFG-/HbA1C+ | | 1.556 |  | 0.527 | 0.003 | 4.739 | 1.688 | 13.306 |
|  | IFG+/HbA1C+ | | 1.697 |  | 0.561 | 0.002 | 5.457 | 1.817 | 16.388 |
| MCV | | | 0.015 |  | 0.028 | 0.584 | 1.016 | 0.961 | 1.073 |
| BMI, body mass index; CI, confidence interval; HbA1C, glycated hemoglobin A1c; HR, hazard ratio; IFG, impaired fasting glucose; kg/m2, kilogram per square meter; MCV, mean corpuscular volume  * BMI criteria by WHO Asia-Pacific criteria  ** (1) IFG-/HbA1c-: FPG < 110.0 mg/dL (6.1 mmol/L) and HbA1c < 6.0% (< 42 mmol/mol); (2) IFG+/HbA1c-: FPG 110–125 mg/dL (6.1–6.9 mmol/L) and HbA1c < 6.0%; (3) HbA1c prediabetes (IFG-/HbA1c+): FPG < 110 mg/dL and HbA1c 6.0%–6.4%; (4) combined IFG and HbA1c prediabetes (IFG+/HbA1c+): FPG 110–125 mg/dL and HbA1c 6.0%–6.4%  *Hazard ratios of diabetic retinopathy were explored with adjustments for age, body mass index, hypertensive status, mean corpuscular volume, sex, and statin use. | | | | | | | | | |
